# The Surgical Management of Pancreatic Pseudocysts: A Narrative Review

**DOI:** 10.7759/cureus.69055

**Published:** 2024-09-10

**Authors:** Ashish Jivani, Raju K Shinde, Tushar Nagtode, Khushbu Vaidya, Somya Goel

**Affiliations:** 1 General Surgery, Jawaharlal Nehru Medical College, Datta Meghe Institute of Higher Education and Research, Wardha, IND

**Keywords:** complications, management strategies, outcomes, pancreatic pseudocysts, surgical techniques

## Abstract

Pancreatic pseudocysts, commonly arising as a complication of acute or chronic pancreatitis, present a significant clinical challenge. This narrative review explores the surgical management of pancreatic pseudocysts, emphasizing advancements, techniques, and outcomes. We examine the indications for surgical intervention, including symptomatic pseudocysts, complications such as infection or hemorrhage, and pseudocysts resistant to conservative treatment. Various surgical approaches are discussed, including open surgery, laparoscopic techniques, and endoscopic interventions. The review highlights the evolution of surgical strategies, from traditional cystogastrostomy to minimally invasive methods, and assesses their efficacy and safety. Additionally, we address patient selection criteria, preoperative assessment, and postoperative care. By synthesizing current evidence and clinical experiences, this review aims to provide a comprehensive overview of the best practices in the surgical management of pancreatic pseudocysts, offering valuable insights for clinicians in optimizing patient outcomes.

## Introduction and background

Pancreatic pseudocysts are common complications of acute or chronic pancreatitis, characterized by localized collections of pancreatic fluid surrounded by a fibrous capsule. These pseudocysts typically result from accumulating digestive enzymes, blood, and necrotic tissue within a well-defined wall formed by granulation tissue. While many pseudocysts resolve spontaneously, others may become symptomatic or lead to significant complications, necessitating surgical intervention [1]. Surgical management of pancreatic pseudocysts has advanced a lot in the last several decades, with minimally invasive techniques complementing conventional approaches in open surgery.

In the past, surgical treatment choices comprised internal drainage procedures like cystogastrostomy and cystoduodenostomy that created a passage between the pseudocyst and the gastrointestinal system for draining cystic contents [2]. However, more recent developments have introduced endoscopic and laparoscopic techniques that offer reduced postoperative morbidity and shorter recovery times [3]. The decision to pursue surgical intervention for pancreatic pseudocysts is guided by several factors, including the size and location of the pseudocyst, the presence of symptoms, and the risk of complications such as infection, bleeding, or obstruction [4]. For instance, pseudocysts larger than 6 cm or those causing symptoms such as abdominal pain or obstruction are more likely to require surgical treatment [5].

This narrative review aims to comprehensively examine the surgical management of pancreatic pseudocysts, focusing on the various surgical techniques available, their indications, outcomes, and the evolving trends in treatment approaches. By synthesizing current evidence and highlighting advancements in surgical techniques, this review seeks to provide a thorough understanding of the optimal management strategies for patients with pancreatic pseudocysts.

## Review

Search methodology

We performed a comprehensive search across multiple databases, including PubMed, Scopus, and Web of Science, covering literature published up to the present. The search strategy utilized a combination of keywords and MeSH terms such as "pancreatic pseudocysts," "surgical management," "surgical treatment," and "drainage." Inclusion criteria focused on original research articles, reviews, and clinical guidelines that discuss surgical interventions for pancreatic pseudocysts, including open surgery, laparoscopic procedures, and endoscopic approaches. Exclusion criteria included studies not available in English and those focusing solely on medical or non-surgical management. The reference lists of relevant articles were manually searched to identify additional pertinent studies. Data extraction focused on surgical techniques, outcomes, complications, and comparisons between different surgical approaches. The results were synthesized to provide a comprehensive overview of the current state of surgical management for pancreatic pseudocysts.

Indications for surgical intervention

Absolute Indications

Infection: Surgical intervention is essential when the pancreatic pseudocyst becomes infected, resulting in a pancreatic abscess or systemic disease. Infected pseudocysts can cause sepsis and require drainage to effectively manage infection and prevent systemic complications. Moreover, infected pseudocysts have high morbidity and mortality rates, and hence surgical treatment cannot be avoided [6].

Hemorrhage: Pseudocysts in the pancreas may burst or erode into the main arteries, leading to blood loss, which calls for surgical operation as unequivocal evidence. In this case, huge internal blood loss can occur, leading to low blood pressure that needs an emergency surgical response to fix the possible ruptures or control the pseudocyst [7].

Relative Indications

Size: Big pseudocysts typically exceeding 6-8 cm could be more likely to cause problems like pressure on neighboring tissues or discomfort. Such large cysts that remain unresolved spontaneously or symptomatic are not the first ones to undergo a surgical operation but would require it nonetheless [8].

Symptoms: Surgery may be indicated in cases where persistent manifestations characterized by abdominal discomfort, nausea or an urge to vomit do not respond to basic methods of treatment. Surgery may be justified if such symptoms substantially interfere with the patient's well-being and fail to improve with other approaches apart from those that involve knife-cutting [4].

Contraindications to Surgery

Severe comorbidities: Patients suffering from noticeably concurrent ailments like extreme heart disease, progressed hepatic issues, or mismanaged diabetes might face severe surgical risks. For patients with a higher risk of surgery than any possible advantages, other methods to manage their conditions must be considered [9].

Uncontrolled infection: Surgical intervention may be contraindicated in cases of pseudocysts alongside uncontrolled or disseminated infection that cannot be treated until the disease is sufficiently controlled [10].

**Figure 1:**
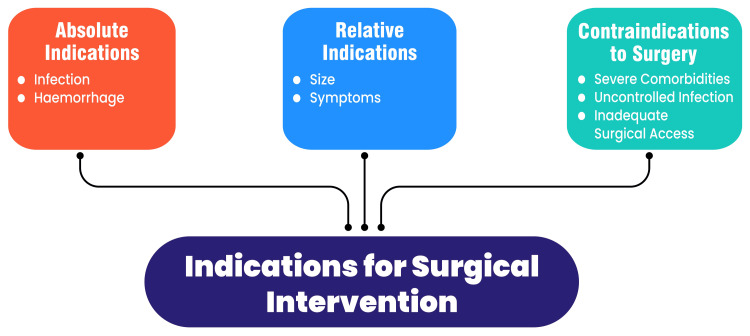
Indications for surgical intervention

Inadequate surgical access: In some cases, anatomical factors or the location of the pseudocyst may make surgical access challenging or risky. Less invasive or alternative interventions may be preferred [6]. Figure 1 presents the indications for surgical intervention.

Surgical approaches to pancreatic pseudocysts

Open Surgical Techniques

Cystogastrostomy: Cystogastrostomy means forming a passage from the pseudocyst to the stomach. Consequently, it enables the emptying of stomach contents. This operation is frequently used for pseudocysts positioned at the back side of the stomach wall. This method is beneficial when tackling extremely massive or symptomatic pseudocysts, as it helps alleviate hiding ailments and prevent possible infections or tearing off [11].

Cystojejunostomy: Cystojejunostomy establishes a link between a pseudocyst and the jejunum, a section of the small intestines. This approach is adopted when the pseudocyst resides in the upper abdominal region or other methods like cystogastrostomy are impossible. Thus, creating this connection, cystic contents are redirected into the jejunum, allowing them to drain and ease the symptoms associated with such conditions [12].

Cystoduodenostomy: Cystoduodenostomy entails establishing a fistula linking the pseudocyst and duodenum - the first segment of the small intestine. This method is applied when there is proximity or contact between the duodenum and pseudocyst. It helps empty the pseudocyst into the duodenum, mixing its contents with digestive enzymes before subsequent processing [13].

External drainage: External drainage involves placing a drainage catheter into the pseudocyst through an abdominal incision, allowing the cyst contents to be continuously evacuated. This technique is often used temporarily or when other surgical options are not feasible [14]. It provides symptomatic relief and reduces the risk of complications related to pseudocyst rupture or infection [15].

Laparoscopic Techniques

Laparoscopic cystogastrostomy: Initially, trocars are inserted into the body through the abdomen to create a pneumoperitoneum. The stomach is mobilized, allowing the anterior wall of the pseudocyst to touch with the gastric wall. A cut is made on the anterior wall of the pseudocyst, followed by a cut on the stomach wall itself. These two incisions are then stitched together, forming an anastomosis. The cyst is drained, and its cavity is inspected for bleeding or leakage [16].

Laparoscopic cystojejunostomy: Pneumoperitoneum can be created, and trocars can be inserted as the first stage of this process. After mobilizing a segment of the jejunum, an opening is made in the pseudocyst, and another cut into the jejunum is required. This way, the two incisions are sewn together so that cyst contents can heap onto the jejunum through an anastomosis-type procedure. Consequently, inspection is done for possible leaks or bleeding at the site [17].

Endoscopic Approaches

Endoscopic ultrasound-guided drainage: The procedure begins with inserting an endoscope equipped with an ultrasound probe into the gastrointestinal tract. Once the pseudocyst is identified, a needle is used to puncture the cyst wall under ultrasound guidance [18]. A catheter or stent is then placed to facilitate continuous drainage of the cystic fluid into the gastrointestinal tract. This method effectively resolves symptoms and reduces pseudocyst size in many patients [19].

Endoscopic cystogastrostomy: This medical process takes place with the help of an endoscope to see the pseudocyst and surrounding stomach area. A needle is put through the endoscope and into the cyst; a stent/catheter is inserted to ensure communication between it and the stomach. The stent will be removed once the pseudocyst has cleared up [20].

Outcomes and complications of surgical management

Success Rates and Long-Term Outcomes

Surgical management of pancreatic pseudocysts generally exhibits favorable outcomes, particularly in appropriately selected patients. Success rates for surgical interventions, such as cystogastrostomy, cystojejunostomy, and cystoduodenostomy, typically range from 80% to 90% [9]. The objective of such processes is to establish a drain via which the gastrointestinal passage is connected with the pseudocyst, thereby alleviating related medical signs and stopping them from recurring in the future. As this is normal, many patients have enjoyed long-term relief from their various ailments and improved living conditions. Another research estimated that the incidence rate for reoccurrence five years after surgical operation stood at about 10%, proving how enduring these actions can be. Unfortunately, however, those managing pancreatic pseudocysts in the future will have to consider potential complications as well as reappearance, which can harm the patient’s overall results [7].

Morbidity and Mortality

The morbidity associated with the surgical management of pancreatic pseudocysts includes both procedure-related complications and long-term health impacts. The overall morbidity rate varies but is often reported between 10% and 30% [21]. Common postoperative complications include infection, pancreatic fistula, and gastrointestinal bleeding. Mortality rates for surgical intervention are relatively low, typically ranging from 1% to 5% [22]. Factors influencing mortality include the patient's overall health status, comorbidities, and the complexity of the pseudocyst. High-risk patients, such as those with advanced pancreatic disease or significant comorbidities, may face higher mortality rates, emphasizing the importance of careful patient selection and preoperative assessment.

Complications

Infection: Postoperative infections, such as wound or intra-abdominal abscesses, are common and may require additional intervention [23].

Pancreatic fistula: This is a significant complication where a pathological connection forms between the pancreatic duct and another organ or the abdominal cavity. It occurs in 5% to 15% of cases and may necessitate additional surgical or conservative management [24].

Advances in surgical management

Minimally Invasive Surgery

Minimally invasive surgery (MIS) has transformed the management of pancreatic pseudocysts, offering advantages such as reduced postoperative pain, shorter recovery times, and fewer complications compared to traditional open surgery. These days, laparoscopic drainage and laparoscopic-assisted procedures are getting increasingly popular. In laparoscopic drainage, small incisions are made on the abdomen through which a laparoscope and specialized instruments are inserted to drain the pseudocyst and, where necessary, to insert internal drains. Studies have shown that laparoscopic procedures can effectively manage pseudocysts, especially those that are not too complicated or large enough [25].

Robotics in Pancreatic Surgery

Pancreatic operations are more precise and can be managed better by robotic procedures, which offer better control in surgery. The da Vinci Surgical System, a robotic system, has been found to improve surgical results by providing three-dimensional images with a better resolution and enabling the delicate handling of surgical instruments. Robotic surgery has been used in cases of pancreatic pseudocysts for both drainage and resection procedures; there is less blood loss after this type of operation than with traditional methods, hence faster patient recovery time. However, the price tag associated with robotic surgery systems remains high, and there’s also a need for special training for these machines [26].

Hybrid Techniques

Hybrid surgical techniques integrate aspects of conventional surgical methods with minimally invasive procedures. These methods are particularly advantageous when solely laparoscopic techniques may not be adequate. For example, a hybrid method may entail laparoscopic entry for the first exploration and drainage; afterward, an open approach might be needed if major complications or anatomical issues arise. The application of hybrid procedures has revealed that they promote flexibility and adaptability of surgical management of pancreatic pseudocysts because they allow individualized treatment based on intra-operative discoveries [27].

Role of Interventional Radiology

Interventional radiology is crucial in managing pancreatic pseudocysts, particularly in non-surgical or pre-surgical contexts. Percutaneous drainage and embolization are two techniques for treating pseudocysts that are hard to reach surgically or to prepare such patients for later surgeries. In percutaneous drainage, a catheter is introduced under imaging guidance to let out the liquid in a way that will relieve symptoms and reduce cyst size before surgical procedures. Interventional radiology’s role in treating pancreatic pseudocysts has broadened, giving more possibilities for patients who may not be suitable candidates for direct surgical methods [28].

Postoperative care and follow-up

Postoperative Management

Pain management: Adequate analgesia is essential for patient comfort and to facilitate early mobilization. Opioids are often used initially, with a gradual transition to non-opioid analgesics as tolerated [29].

Nutritional support: Nutritional status should be closely monitored. Enteral feeding is preferred when feasible, but parenteral nutrition may be necessary in the initial postoperative period if gastrointestinal function is impaired [30].

Wound care: Regular inspection and care of the surgical wound are vital to prevent infections. Drainage tubes, if placed, should be monitored for signs of leakage or infection, and their output should be carefully documented [31].

Early mobilization: Encouraging early mobilization reduces the risk of deep vein thrombosis (DVT) and improves overall recovery. This should be tailored to the patient’s condition and pain levels [32].

Management of complications: Postoperative complications such as pancreatic fistula, intra-abdominal abscess, or bleeding should be promptly addressed. Regular imaging and laboratory tests help in the early detection and management of such issues [9].

Monitoring for Recurrence

Imaging studies: Routine follow-up imaging, such as abdominal ultrasound or computed tomography (CT) scans, is recommended to detect any recurrence or complications. The imaging frequency is typically determined by the initial cyst characteristics and the surgical outcome [33].

Clinical evaluation: Regular clinical evaluations are essential for assessing symptoms that might suggest recurrences, such as abdominal pain or discomfort. A thorough clinical history and physical examination complement imaging studies [34].

Endoscopy: In some cases, endoscopic evaluation may be necessary if symptoms persist or if imaging results are inconclusive. Endoscopic ultrasound (EUS) can provide additional information about the status of the pseudocyst [35].

Biomarkers: Although not routinely used, biomarkers such as serum amylase and lipase levels can be monitored in specific cases to help assess pancreatic function and potential complications [36].

Long-Term Outcomes and Quality of Life

Recovery and function: Most patients experience significant improvement in symptoms following successful surgical management. However, some may develop long-term sequelae such as pancreatic insufficiency or diabetes, which requires ongoing management [37].

Quality of life: Studies have shown that patients who undergo successful surgical intervention for pancreatic pseudocysts often report improved quality of life compared to those with untreated pseudocysts. Psychological support and counseling may be beneficial, especially for those dealing with chronic pain or other long-term effects [38].

Long-term surveillance: Regular follow-up visits are essential to monitor for delayed complications or recurrence. Patient education regarding symptom management and lifestyle modifications is also important for maintaining long-term health and well-being [4].

Patient-reported outcomes: Incorporating patient-reported outcome measures (PROMs) into follow-up care can provide valuable insights into the impact of the surgery on daily life and help tailor ongoing care [39].

Discussion

In the surgical management of pancreatic pseudocysts, various approaches have been developed to address both absolute and relative indications for intervention, such as infection, bleeding, and symptomatic cysts. Traditional open surgery has been largely supplemented by minimally invasive techniques, including laparoscopic and endoscopic procedures, which offer benefits such as reduced recovery times and lower postoperative complication rates [40]. However, the choice of surgical approach often depends on factors like the cyst's size, location, and the patient’s overall health. While these techniques generally yield positive outcomes, complications such as pancreatic fistulas and infections remain significant concerns, emphasizing the need for careful patient selection and surgical planning [31].

Even though surgical techniques have progressed, there are still challenges, especially when it comes to standardizing treatment protocols because variability in results is determined by the surgeon’s skill and patient condition. Furthermore, one constraint on current literature is that it lacks long-term data; thus, assessing different surgical strategies for effectiveness is difficult [33]. Future research should focus on refining minimally invasive techniques, enhancing personalized treatment approaches, and conducting long-term studies to better inform clinical guidelines. Additionally, integrating multidisciplinary care teams could further optimize the management of pancreatic pseudocysts, leading to improved patient outcomes [12].

Future directions in surgical management

Future directions in the surgical management of pancreatic pseudocysts should focus on advancing minimally invasive techniques to reduce complication rates and improve patient outcomes. The emergence of robotics in surgery may make it more accurate and reduce its complexities. Moreover, it implies that modern imaging devices can lead us to personalized and localized interventions. In addition, relevant studies should be done over a longer period on different kinds of surgeries to understand how long they last and whether they are effective in their use. This could help develop clearer guidelines regarding patient treatment options. Finally, multidisciplinary care models based on cooperation between surgeons, radiologists, and gastroenterologists may improve the quality of patient treatment in terms of pancreatic pseudocyst management.

## Conclusions

The surgical management of pancreatic pseudocysts remains a critical area of clinical practice, particularly when complications necessitate intervention. This narrative review highlights the significant progress made in the field, particularly with the advent of minimally invasive techniques that have improved patient outcomes by reducing recovery time and postoperative complications. However, challenges persist, including the variability in surgical outcomes, the risk of complications, and the lack of long-term data to guide treatment decisions. Future efforts should focus on refining surgical techniques, expanding our understanding of long-term outcomes, and personalizing treatment strategies to better align with individual patient needs. Additionally, a multidisciplinary approach to management involving collaboration between surgeons, radiologists, and gastroenterologists could further enhance patient care and optimize surgical outcomes. While the surgical management of pancreatic pseudocysts has evolved significantly, ongoing research and innovation are key to addressing current limitations and improving the quality of care for these patients.
